# Socioeconomic Disparities and In-Hospital Outcomes in Acute Myocardial Infarction: A Case-Control Study

**DOI:** 10.7759/cureus.83551

**Published:** 2025-05-06

**Authors:** Eman Shaban, Eman Khashaba, Ensaf Bassam, Ayman A Abdelaziz, Amira Shaban, Ahmed Shaban, Hany A Zaki

**Affiliations:** 1 Cardiology, Al Jufairi Diagnostic And Treatment, Doha, QAT; 2 Public Health and Community, Faculty of Medicine, Mansoura University, Mansoura, EGY; 3 Cardiovascular Medicine, Faculty of Medicine, Mansoura University, Mansoura, EGY; 4 Internal Medicine, Mansoura University Hospitals, Mansoura, EGY; 5 Medicine, Faculty of Medicine, Mansoura University, Mansoura, EGY; 6 Emergency Medicine, Qatar University College of Medicine, Doha, QAT

**Keywords:** case-control study, heart, in-hospital outcome, myocardial infarction, socioeconomic status (ses)

## Abstract

Purpose: This study aimed to assess the impact of socioeconomic status (SES) primarily on in-hospital outcomes, while also exploring its association with the incidence of acute myocardial infarction (AMI).

Methods: This was a case-control study that included 100 patients with first-onset AMI and 100 age- and sex-matched controls without clinical or investigative evidence of cardiac disease, confirmed by history, ECG, and absence of prior hospitalizations. Data collection involved demographics, cardiovascular risk factors (e.g., smoking, obesity, hypertension), blood pressure, BMI, echocardiography, and laboratory investigations, used diagnostically and prognostically. SES was assessed at admission using the Egyptian socioeconomic scale (total score: 84), covering seven domains. SES was categorized into very low (<40), low (40-56.9), middle (57-64.9), and high (>65). Assessing SES before outcome measurement reduced reverse causation risk.

Results: Occurrences and in-hospital outcomes, such as cardiogenic shock, were significantly more common among AMI patients from lower SES groups compared to higher SES groups (p < 0.05). Mortality was also higher in the lower SES group, with an odds ratio of 4.8 (95% confidence interval (CI): 1.5-16.6), indicating a more than fourfold increased risk. However, the wide CI suggests some uncertainty in the estimate, likely due to the limited sample size. In-hospital complications were reported in 41.1% of patients with low and very low SES (39 patients), compared to 20.4% (21 patients) and 32.7% (32 patients) in the middle and high SES groups, respectively.

Conclusion: These findings highlight that SES significantly shapes in-hospital outcomes among patients with first-onset AMI. Patients from lower SES groups experienced more frequent complications and higher mortality. While comorbid conditions such as hypertension, diabetes, and obesity were more prevalent in lower SES groups, SES itself served as the primary exposure variable influencing outcomes, rather than being evaluated through the presence of comorbidities.

## Introduction

Myocardial infarction (MI) is a principal contributor to both morbidity and mortality rates globally and is defined as myocardial cell death caused by prolonged ischemia [1]. The most common underlying cause of MI is an acute thrombus obstructing an atherosclerotic coronary artery [2]. Many risk factors are associated with acute MI, including tobacco use, hyperlipidemia, hypertension, diabetes, obesity, male gender, and family history [3-7]. These factors increase cardiovascular risk through various mechanisms, such as promoting atherosclerosis or impairing vascular function. For example, smoking induces endothelial injury and increases platelet aggregation, while hypertension accelerates arterial wall damage and atherosclerotic plaque formation [8,9].

Among these risk factors, socioeconomic status (SES) has increasingly been recognized as a significant contributor to both the occurrence and outcomes of MI [10]. Individuals with low SES often live in socioeconomically disadvantaged neighborhoods, where there is a higher prevalence of smoking, poor dietary habits, physical inactivity, and limited access to healthcare services. These factors contribute substantially to the increased cardiovascular burden observed in these populations [11]. In addition, low SES is associated with lower rates of treatment adherence and self-management, which can worsen prognosis following acute events like MI [12]. Prolonged socioeconomic disadvantage, such as low parental SES, limited education, and low personal income, has also been associated with a heightened risk of MI and adverse outcomes [13-15].

Even though high-quality evidence supports the notion that low SES increases the risk of MI [16] and worsens hospital outcomes in MI patients [17], there is a scarcity of literature that explicitly assesses the association between SES and MI occurrence and in-hospital outcomes in Egypt. Existing studies, if any, are often limited in scope, methodology, or population coverage. Therefore, this study aimed to evaluate the association between SES and the occurrence of acute MI and in-hospital outcomes among AMI patients, such as hospital mortality and complications. 

This article was previously posted to the Research Square preprint server on July 2, 2024 (https://doi.org/10.21203/rs.3.rs-4602022/v1).

## Materials and methods

This was an observational and analytical case-control study conducted in the Cardiology Department, specifically within the Coronary Care Unit (CCU), of the Specialized Medical Hospital, Mansoura University, Mansoura, Egypt. The study focused on patients experiencing their first AMI between January 2015 and February 2016. The Medical Research Ethics Committee, Mansoura University, approved the study (approval number: MS/758).

Study population

A total of 100 patients admitted with a first-onset AMI (cases) and 100 age- and sex-matched controls without a history or evidence of ischemic heart disease were included in the study. The majority of the case group were married and aged ≥41 years. Controls were recruited from employees at Mansoura University Hospitals. They underwent screening through medical history, physical examination, electrocardiography (ECG), and laboratory tests to ensure the absence of MI, angina, or structural heart disease.

Inclusion Criteria

Patients aged ≥18 who presented with new-onset acute MI and were initially admitted to the CCU were eligible for inclusion.

Exclusion Criteria

Patients were excluded if they had a prior history of MI, unstable angina, non-ST-segment elevation MI, rheumatic valvular heart disease, congenital heart disease, malignancy, chronic liver disease, or chronic renal failure. Controls with major cardiovascular risk factors were excluded to reduce residual confounding.

Data collection

Data were collected prospectively during admission through structured history-taking and clinical examination. This included demographic details, anthropometric measurements, and cardiovascular risk factors (e.g., smoking, hypertension, diabetes, dyslipidemia, and obesity). BMI was calculated as weight (kg) divided by height squared (m²) and classified according to WHO guidelines: underweight (<18.5), normal (18.5-24.9), overweight (pre-obesity) (25.0-29.9), and obesity (≥30) [18].

Clinical and laboratory investigations included blood pressure, random blood glucose, serum creatinine, fasting lipid profile, troponin I, creatine phosphokinase (CPK), creatine kinase-myocardial band (CK-MB), ECG, and echocardiography. Patients were followed throughout hospitalization (4-10 days). In-hospital outcomes assessed were cardiogenic shock, arrhythmia, reinfarction, heart failure, and death. These outcomes were diagnosed by the attending physicians based on clinical criteria, imaging, and lab findings, and recorded in patient charts. SES data were collected at admission using a validated Egyptian Socioeconomic Scale that includes seven domains (education, occupation, family possessions, household characteristics, sanitation, economic status, and healthcare access) with a total score of 84 [19]. Based on score quartiles, SES was categorized as very low (<40), low (40-56.9), middle (57-64.9), and high (>65). Data collectors were blinded to patient outcomes, and outcome assessors were blinded to SES classification to minimize detection bias.

Statistical analysis

A formal sample size calculation was not performed; however, the sample size was deemed adequate based on feasibility and prior similar studies. Data were analyzed using IBM SPSS Statistics for Windows, Version 20.0 (Released 2011; IBM Corp., Armonk, New York, United States). Normality was tested using the one-sample Kolmogorov-Smirnov test. Continuous parametric variables were reported as mean ± standard deviation (SD) and compared using the independent t-test. Non-parametric data were summarized using median (range) and analyzed with the Mann-Whitney U test. Categorical variables were expressed as frequencies and percentages, and differences were assessed using Pearson’s Chi-square test. In-hospital outcomes were compared across SES groups. A p-value <0.05 was considered statistically significant. Potential confounders such as smoking, BMI, diabetes, and hypertension were considered in the interpretation; however, no multivariable analysis was conducted due to sample size limitations.

## Results

Nearly half of the participants were between 41 and 60 years of age (n = 100, 50%). The majority of both cases and controls resided in urban areas (cases: 59%, n = 59; controls: 63%, n = 63). Additionally, most participants were married (cases: 97%, n = 97; controls: 100%, n = 100) (Table 1).

**Table 1:** Sociodemographic characteristics among study groups

Sociodemographic characteristics	Cases (N=100)	Control (N=100)	Chi-square test	p value
	Frequency (Percentage)	Frequency (Percentage)		
Age groups (years)				
≤40 years	23 (23)	22 (22)	0.1	0.9
41-60	51 (51)	50 (50)		
≥61	26 (26)	28 (28)		
Sex				
Male	68 (68)	68 (68)	--	1.0
Female	32 (32)	32 (32)		
Residence				
Urban	59 (59)	63 (63)	0.3	0.5
Rural	41 (41)	37 (37)		
Marital status				
Single	0	3 (3)	3.04	0.2
Married	100 (100)	97 (97)		

There was no statistically significant difference between the case and control groups regarding socioeconomic categories. The mean socioeconomic score was slightly higher among the controls (53.3 ± 14.7) compared to the cases (51.2 ± 15.2), although this difference was not statistically significant (p > 0.05) (Table 2).

**Table 2:** Comparison of socioeconomic score categories among study groups

Socioeconomic categories	Cases (N=100)	Control (N=100)	Significance test	p-value OR (95%CI)
	Frequency (Percentage)	Frequency (Percentage)		
Very low <40	25 (25)	25 (25)	0.15	0.6 1.17 (0.5-2.7)
Low 40-56.9	32 (32)	17 (17)	3.7	0.05 2.2 (0.9-5.3)
Middle 57-64.9	19 (19)	30 (30)	0.3	0.5 0.74 (0.3-1.76)
High (r) >65	24 (24)	28 (28)	-	
Total score (Mean±SD)	51.2±15.2	53.3 ±14.7	1.02	0.3

The mean BMI was significantly higher in the case group compared to the control group (p < 0.05). The frequency of heavy smoking was notably greater among cases, demonstrating a statistically significant association and an approximately 11-fold increased risk (p < 0.05, OR = 11.8). Similarly, hypertension was more prevalent in the case group, with a significant difference and a 13-fold increased risk (p < 0.05, OR = 13.57). In addition, the prevalence of diabetes mellitus was significantly higher among cases compared to controls, with an OR of 2.69 (p < 0.05). The occurrence of dyslipidemia was also elevated in the case group, showing a statistically significant difference relative to the control group (p < 0.05) (Table 3).

**Table 3:** Associated factors with first acute myocardial infarction among the study groups χ2:  Chi-square test; ◊ t: independent t-test; *p-value is statistically significant when<0.05

Risk factors	Cases (N=100)	Control (N=100)	Test of significance	p value	OR (95% CI)
	Frequency (Percentage)	Frequency (Percentage)			
Smoking					
Nonsmoker (r)	45 (45)	79 (79)			
Smokers	22 (22)	4 (4)	χ^2^=20.17	<0.0001*	9.6 (2.9-35.5)
Ex-smoker	6 (6)	13 (13)	χ^2^=0.16	0.6	0.81(0.2-2.5)
Heavy smoker	27 (27)	4 (4)	χ^2^=25.5	<0.0001*	11.8(3.6-42.8)
Hypertension	34 (34)	3 (3)	χ^2^=31.87	<0.001*	13.6 (3.9-46.2)
Diabetes	21 (21)	9 (9)	χ^2^=5.65	0.017*	2.7 (1.2-6.2)
Dyslipidemia	59 (59)	2 (2)	χ^2^=76.64	<0.0001*	70.5 (16.4-302.3)
◊BMI (mean±SD)	33.3±8.5	28.7 ±3.9	t=4.8	<0.0001*	

Additionally, the incidence of cardiogenic shock was significantly higher among patients in the lower SES group compared to those in the higher SES group (p < 0.05), highlighting a notable disparity. This finding emphasizes the impact of SES on the occurrence of in-hospital complications. Furthermore, mortality rates were significantly elevated in the lower SES group, with a fourfold increased risk compared to the higher SES group (OR = 4.8; 95%CI: 1.5-16.6) (Table 4).

**Table 4:** In-hospital complications according to socioeconomic status * Statistically significant result at p < 0.05.

Outcome	Median socioeconomic score		Chi-square test	p-value	OR (95% CI)
	<57, Lower SES (n=51), n (%)	≥57, Higher SES (n=49), n (%)			
Pulmonary edema					
Yes	11 (21.6)	8 (16.3)	0.4	0.5	-
No	40 (78.4)	41 (83.7)			
Cardiogenic shock					
Yes	19 (37.3)	8 (16.3)	5.5	0.01*	3.04 (1.08-8.7)
No	32 (62.7)	41 (83.7)			
Arrhythmia					
Yes	17 (33.3)	14 (28.6)	0.26	0.6	-----
No	34 (66.7)	35 (71.4)			
Death					
Yes	18 (35.3)	5 (10.2)	8.7	0.003*	4.8 (1.5-16.6)
No	33 (64.7)	44 (89.8)			

Additionally, in-hospital complications were more frequent among patients with low and very low SES, occurring in 23 out of 57 cases (41.1%), compared to four out of 19 cases (21.1%) in the middle SES group and eight out of 24 cases (33.3%) in the high SES group. Mortality was also higher in the low and very low SES groups, reported in 19 out of 57 cases (33.3%), compared to four out of 19 cases (21.1%) in the middle SES group, and was absent in the high SES group (Table 5).

**Table 5:** Bivariate analysis for adverse outcomes related to socioeconomic status P#: comparison between three groups of SES; P1: low and very low versus high groups of SES; P2: middle versus high groups of SES * Statistically significant result at p < 0.05. SES: socioeconomic status

Variables	Low and very low SES (n=57)	Middle SES (n=19)	High SES (n=24)	P-value	OR (95% CI)
	Frequency (Percentage)	Frequency (Percentage)	Frequency (Percentage)		
In-hospital complications	41 (41.4)	10 (20.4)	17 (32.7)	P#0.03*	1.06 (0.32-3.3)
				P1=0.9	
				P2=0.2	0.46 (0.11-1.9)
Death	19 (33.9)	4 (21.1)	0 (0)	P# 0.001*	undefined
				P1=0.001*	
				P2=0.01*	

A statistically significant difference was observed in the number of patients receiving percutaneous coronary intervention (PCI), with 35 out of 49 patients (71.4%) in the higher SES group undergoing PCI compared to 14 out of 51 patients (27.5%) in the lower SES group (p < 0.001). Although streptokinase therapy was more frequently administered to patients in the lower SES group (34 out of 51, 66.7%) than those in the higher SES group (11 out of 49, 22.4%), this difference was not statistically significant (p > 0.05). Additionally, the median time to seeking medical care was significantly longer in the lower SES group compared to the higher SES group (p < 0.001) (Table 6).

**Table 6:** Effect of SES on type of chest pain, mode of treatment, and time till seeking medical care * Statistically significant result at p < 0.05. SES: socioeconomic status; PCI: percutaneous coronary intervention

	Median Socioeconomic Score		P-value	In-hospital complication		P value
	<57, Lower SES (n=51), n (%)	≥57, Higher SES (n=49), n (%)		No	Yes	
Chest pain						
Typical	36 (71.6)	35 (71.4)	0.9	24(75)	47(96.1)	0.6
Atypical	15 (29.4)	14 (28.6)		8(25)	21(30.9)	
PCI treatment						
Yes	14 (27.5)	35 (71.4)	<0.001*	37(67.3)	12(26.7)	<0.001*
No	37 (72.5)	14 (28.6)		18(32.7)	33(73.3)	
Streptokinase therapy						
yes	21 (41.2)	12 (24.5)	0.07	12(37.5)	21(30.9)	0.5
No	30 (58.8)	37 (75.5)		20(62.5)	47(69.1)	
Time till seeking medical care (days)						
Median	7	5	<0.001*	5	6	0.02*
Min-max	0-48	0-14		0-48	0-18	

**Figure 1:**
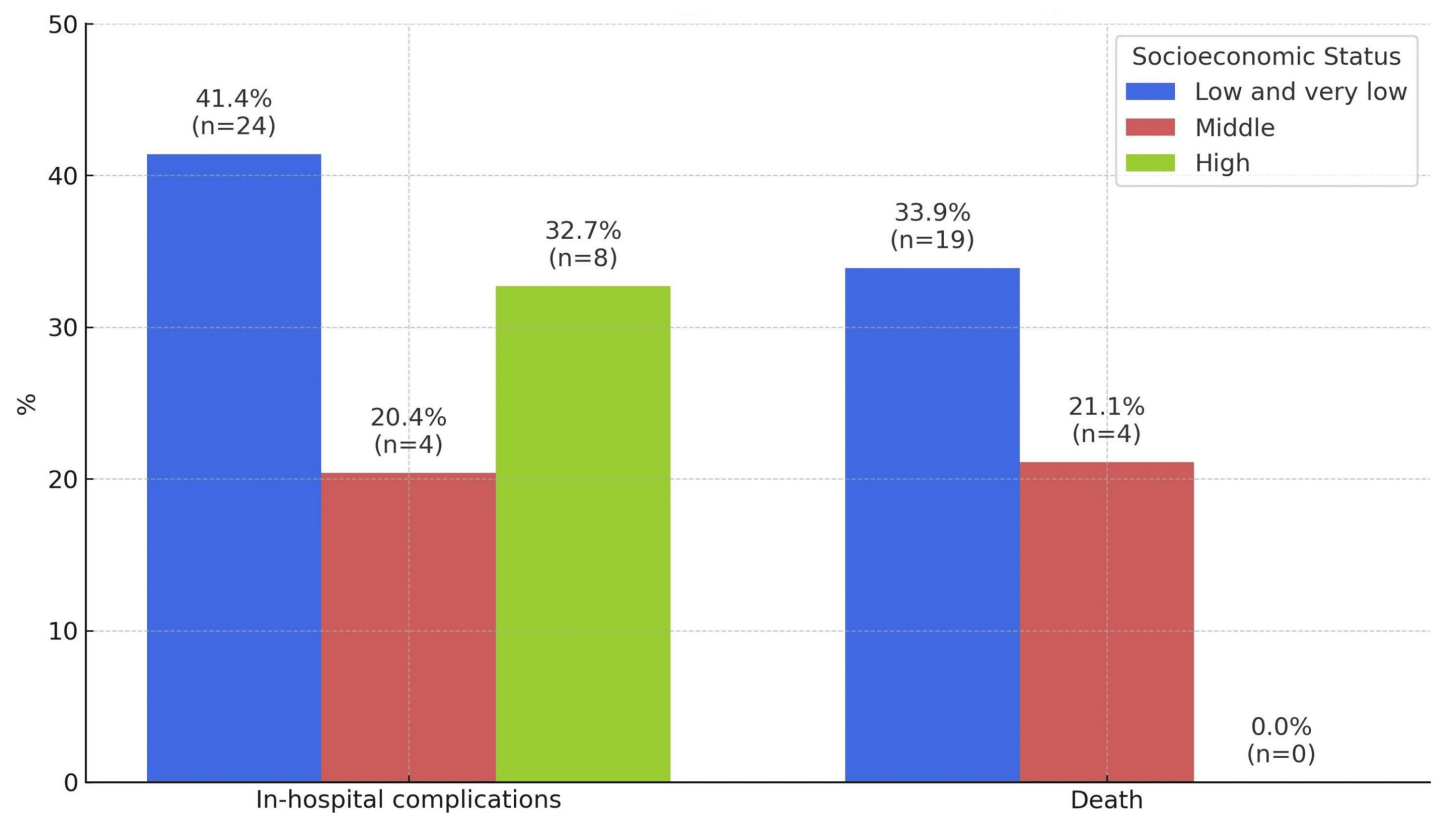
In-hospital complications and mortality stratified by socioeconomic status

When in-hospital outcomes were analyzed with SES, patients in the low and very low SES groups experienced complications more frequently, occurring in 23 out of 57 cases (41.1%), compared to four out of 19 cases (20.4%) in the middle SES group and eight out of 24 cases (32.7%) in the high SES group. Similarly, mortality was higher among the low and very low SES groups, reported in 19 out of 57 cases (33.3%), compared to four out of 19 cases (21.1%) in the middle SES group, and was absent in the high SES group (Figure 1).

## Discussion

The primary aim of the present study was to assess the impact of SES on the incidence of AMI and its in-hospital outcomes, including complications such as arrhythmias, cardiogenic shock, mechanical complications, pulmonary edema, bleeding, stroke, and mortality during hospital stay. The study also aimed to explore the relationship between SES and the first occurrence of MI.

The findings of this study suggest a significant association between low SES and increased occurrence of AMI. This is consistent with several previous studies that reported a link between lower SES and higher incidence of MI [20-22]. However, other studies have shown conflicting results, where a higher prevalence of cardiovascular disease was observed among individuals in middle or high SES groups [23,24]. Various factors, including differences in population demographics, access to healthcare, diagnostic practices, and distribution of risk factors, may explain these differences. While most large-scale studies support the association between low SES and increased MI risk, inconsistencies in some individual studies highlight the need for further well-designed, context-specific research.

The present study showed that individuals from lower SES backgrounds had a higher prevalence of key cardiovascular risk factors, including abdominal obesity, dyslipidemia, smoking, hypertension, and diabetes mellitus. These findings are consistent with existing literature indicating that health-related behaviors, such as smoking, physical inactivity, poor diet, and low adherence to medical care, are more common among individuals in lower socioeconomic groups [25-29]. These behaviors are linked to the development of cardiovascular diseases such as hypertension and coronary artery disease. Previous research estimated that approximately 50% of the SES-related differences in MI risk could be explained by four major modifiable risk factors: hypertension, smoking, high cholesterol, and diabetes [30,31].

In this study, cardiogenic shock and in-hospital complications were more frequent among low-SES patients compared to those from higher SES categories. Additionally, mortality during hospitalization was higher among the lower SES group. These results align with earlier studies that reported increased acute and long-term mortality and complications after MI among patients in lower socioeconomic strata [32-34]. One explanation could be that lower SES patients are less likely to receive timely and aggressive treatment during hospitalization, which may contribute to worse outcomes. Several studies have demonstrated that the absence of health insurance or being part of a disadvantaged group can lead to limited access to advanced treatments, such as PCI, and consequently to increased mortality [33,34].

Despite improvements in cardiovascular care globally, morbidity and mortality due to cardiovascular diseases remain high in developing countries, including Egypt. Previous studies have reported MI to be one of the leading causes of death in the Egyptian population [35,36]. The current study provides important insights into the Egyptian context, demonstrating that individuals with low SES are more likely to develop MI and suffer from poorer outcomes. This may be due to several factors, including lack of awareness, limited education, delayed medical attention, and restricted access to optimal treatment. Higher SES individuals often have better knowledge about early symptoms and access to timely healthcare, which may lead to more favorable outcomes [37,38].

Age appeared to be another critical factor in this study. A greater proportion of low-SES patients were older than 41 than those in the higher SES group. This observation is supported by prior research, which reported that premature MI tends to affect more socioeconomically deprived individuals [39]. Furthermore, increased case fatality with age may be attributed to reduced effectiveness of life-saving treatments, delayed hospitalization, lower procedure utilization, and increased comorbidities among the elderly. On the other hand, high SES may carry a greater relative risk in younger age groups due to specific lifestyle and behavioral patterns [40,41]. Socioeconomic disparities in cardiovascular risk factors, mainly smoking, are often more prominent in younger individuals [42,43].

In our study, PCI was significantly more common in patients from higher SES backgrounds. At the same time, streptokinase use was more prevalent among those with low SES, likely due to its availability through governmental programs. Furthermore, the low-SES group had a longer delay in seeking medical care after the onset of symptoms. These findings are consistent with previous reports showing that lower SES patients often arrive later at the emergency department and are less likely to receive advanced interventions due to financial or systemic barriers [44-46]. Studies also show that the lack of rapid and appropriate emergency care contributes to poor outcomes following MI, particularly among socioeconomically disadvantaged individuals [47,48].

Limitations

This study has several limitations. It was conducted at a single center and included only patients admitted to the hospital for AMI, excluding those who died before arrival or were treated at other facilities. The study also focused only on in-hospital outcomes and did not include follow-up data after discharge; therefore, long-term treatment adherence and outcomes remain unknown. In addition, while the study explored associations between SES and outcomes, no multivariate analysis was performed to adjust for confounding factors such as age, sex, or comorbidities. The generalizability of findings may be limited to the Mansoura region. Finally, the study did not assess whether the association between SES and outcomes differed by sex, despite well-established gender differences in MI presentation and treatment. Future studies should examine this interaction to gain deeper insight.

## Conclusions

This study demonstrates a significant association between low SES and both the incidence of AMI and adverse in-hospital outcomes. Patients from lower SES backgrounds exhibited a higher prevalence of cardiovascular risk factors, including smoking, hypertension, diabetes, dyslipidemia, and obesity, as well as delays in seeking medical care. These factors likely contributed to the increased rates of complications, cardiogenic shock, and mortality observed in this group. Disparities in management were also evident, with higher SES patients more likely to receive percutaneous coronary intervention, while lower SES patients more often received streptokinase. However, the latter difference was not statistically significant.

While the findings demonstrate associations rather than causation, they underscore the critical impact of SES on AMI presentation, management, and outcomes. These results highlight the need for targeted interventions, including early risk screening, public health education, and equitable access to care for underserved populations. Since only in-hospital outcomes were assessed, future studies should examine long-term outcomes, follow-up care disparities, and the consistency of these associations across demographic subgroups such as age and sex.
